# The effect of mirabegron, used for overactive bladder treatment, on female sexual function: a prospective controlled study

**DOI:** 10.1186/s12894-018-0377-9

**Published:** 2018-06-25

**Authors:** A. Zachariou, C. Mamoulakis, M. Filiponi, F. Dimitriadis, J. Giannakis, S. Skouros, P. Tsounapi, A. Takenaka, N. Sofikitis

**Affiliations:** 1 Department of Urology, School of Medicine, Ioannina University, Ioannina, Greece; 2 Department of Urology, University General Hospital of Heraklion, University of Crete, Medical School, Heraklion, Greece; 3 Department of Urology, ELPIS Hospital, Volos, Greece; 4 Department of Urology, School of Medicine, Tottori University, Yonago, Japan; 5 3 Spyridi Street, 38221 Volos, Greece

**Keywords:** Female sexual dysfunction, Mirabegron, Overactive bladder

## Abstract

**Background:**

Αim of the study was to determine the effect of mirabegron, used for overactive bladder (OAB) treatment, on female sexual function.

**Methods:**

Eighty five sexually active women suffering from overactive bladder were prospectively enrolled in this study. Females were divided into two groups. In Group A (control), 48 patients received no treatment and in Group B, 37 patients received mirabegron 50 mg/daily for 3 months. Patients were evaluated with FSFI-Gr at the beginning of the study and again after a period of 3 months.

**Results:**

In Group B, there was a significant increase post-treatment compared to baseline (*p* < 0.001) in total FSFI (20.3 (3.8) to 26.6 (4.2)) and all domains (desire: 3.0 (1.2) to 4.8 (1.2)), arousal: 3.0 (0.8) to 4.8 (0.9), lubrication: 3.9 (1.1) to 4.8 (1.2), orgasm: 3.6 (0.8) to 4.8 (1.0), satisfaction: 3.2 (0.4) to 4.0 (0.8) and pain: 3.2 (0.8) to 4.4 (1.2)). In Group A, there were no statistically significant changes in pre- and post-observation values.

**Conclusions:**

This study is one of the few demonstrating that management of OAB with mirabegron improves female sexual function.

**Trial registration:**

TRN ISRCTN17199301, 20/10/2017, retrospectively registered.

## Background

Overactive bladder (OAB) is defined by the International Continence Society (ICS) as urinary urgency in the absence of any known infection or other obvious pathology. OAB is usually characterized by frequency and nocturia, but may or may not cause urinary incontinence [1]. It has been shown to affect up to 36% of adult women in Europe and US [2, 3].

Although not life threatening, OAB is a debilitating disease which can substantially impede the quality of life, resulting in low self-esteem, anxiety, depression, impairment of work productivity and increase in the number of falls and fractures [4]. Women with OAB experience increased incidence of sexual problems, sometimes with consequent personal distress and sexual partner compatibility issues. [5]. The impact of OAB symptoms on sexual function in women has been evaluated in a few studies [6–9]. Patel et al. [6] reported that 25% of their female OAB population had some degree of sexual dysfunction, meaning that OAB has a greater effect on female sexual health than it does urinary incontinence.

Female sexual dysfunction (FSD) is traditionally classified into disorders of desire, arousal, lubrication, orgasm and pain. In the absence of detailed epidemiological data, current estimates have up to 43% of women complaining of at least one sexual issue [10]. Women are at risk of developing FSD due to physiologic, iatrogenic and psychological factors. Lower tract urinary tract infections are a further, independent FSD cause [10, 11]. To identify FSD, appropriate assessment guidelines should be applied. So as to ascertain sexual history and enable assessment, there are a number of self-reporting questionnaires available. The Female Sexual Function Index (FSFI) is a concise, multidimensional “gold standard” tool which is regarded in high-esteem [12].

Recently introduced as an oral treatment for OAB, mirabegron (a β3-adrenergic agonist compound) improves storage capacity of bladder without inducing anticholinergic adverse events [13]. In four, large–scale 12-week phase III studies [14–17], a pooled analysis [18] and 12-month study [19], mirabegron consistently demonstrated superiority over placebo with respect to reductions in incontinence episodes and micturition frequency, with a similar incidence of adverse effects as the placebo.

The objective of the current study is to evaluate the effect of the β3-adrenoceptor agonist, mirabegron, as used for OAB treatment on the sexual function of women (employing FSFI-Gr, a validated questionnaire translated into the Greek language).

## Methods

Between January 2016 and December 2016, 85 sexually active women with confirmed OAB, had referred to the Urogynecology outpatient clinic and were prospectively enrolled in this study. OAB was determined using the International Continence Society definition [1]. The urination frequency of all women was 8 or more times a day, with urge symptoms independent of incontinence. OAB was present in all women for a minimum of 3 months and none of the women had undergone prior treatment for the condition. Other inclusion criteria were the willing of women to comply with the protocol and the capability to complete the voiding diaries and the questionnaires without assistance.

Subjects were excluded from inclusion if they had clinically significant stress urinary incontinence, neurogenic bladder and urinary retention or were at risk of these conditions. Women with a history of pelvic muscle training programs were excluded because it is accepted that pelvic floor muscle exercises improve female sexual function [20]. The protocol for the research project was approved by ELPIS HOSPITAL Ethics Committee and informed consent was taken from all the women.

The inclusion criteria also integrated women over the age of 18 being in a sexually active relationship. Women who stated that they were not sexually active were asked to indicate the reason and were excluded from further analysis. Women who considered that there was no need for long-term treatment for OAB or were afraid of regimen’s adverse effects were included in control group.

Patients were assessed using a comprehensive history, a detailed general and neurological physical examination, as well FSFI. According to the latest report of the International Consultation on Sexual Medicine, the FSFI remains the “gold standard” assessment tool, has a level of evidence 1 and recommendation grade A, for evaluating female sexual dysfunction [21]. Item selection and categories were based on the American Foundation for Urological Diseases classification system of female sexual dysfunction. Furthermore, FSFI has been translated and validated in the Greek language [22]. All women were asked to complete the Greek version of the FSFI, which evaluates the four phases of female sexual function and categorizes sexual dysfunction in the domains of (a) desire (b) arousal (c) lubrication (d) orgasm (e) satisfaction and (f) pain. The development of a scoring system, whereby higher scores indicate a healthier condition allowed the attainment of individual domain scores. Wiegel et al. [23] found that a total FSFI score of 26.5 is the optimal cut-off score for differentiating women with and without sexual dysfunction. We used the same cut-off scores in order to have comparable results with matching papers.

To determine the eligible women all females were asked to answer the question: “Do you have sexual distress associated with sexual dysfunction?” and only women who gave a negative answer were finally recruited for analysis, since sexual distress needs special questionnaires to be evaluated. Patients were divided into two groups. Group A, which is defined as a control group, consisted of 48 women. None of these females with OAB wished to receive any therapy. On the other hand, in Group B, 37 patients with OAB were treated with mirabegron 50 mg daily for 3 months.

Patients of Group A (without OAB therapy) completed a 3 day micturition diary prior to and after 3 month-observation-period. Patients of Group B completed a 3 day micturition diary prior to and immediately after the third month of mirabegron treatment. For each episode of urinary symptoms, the patient recorded the date and time, regardless of the presence of urgency and/or incontinence, the volume voided and the influence of the episode (of urinary symptoms) on the patient’s sleep. All women within Group B attended monthly office visits to ensure patients’ compliance with the treatment. In Group A, all women attended monthly office visits to ensure that they did not have any pharmacotherapy or behavioral therapy for OAB. Within Group A and Group B, voiding frequency, nocturia, urgency episodes, incontinent episodes, number of incontinence pads used, and voided volume were measured post-treatment using the 3 day micturition diary. All patients completed the FSFI questionnaire at the beginning and after the completion of the 3 month study.

All outcome variables were tested for normality using the Shapiro-Wilk W test. Concretely, the following continues/ordinal variables were tested per group: age, body weight, symptom duration, parity; pre- and post-observation/treatment FSFI domains (desire, arousal, lubrication, orgasm, satisfaction, pain), total score of sexual function and percentage (%) improvement in total score of sexual function; pre- and post-observation/treatment episodes of frequency, urgency, nocturia, incontinence; number of pads needed; and voided volumes. The potential presence of any correlation was investigated between the % of improvement in LUTS/incontinence and in sexual function (separately for each parameter) in order to evaluate the effect of OAB improvement on sexual function. Finally, % improvement in total score of sexual function was compared between groups and a multivariate linear regression analysis was performed in order to investigate the effect of several independent parameters on sexual function. The % improvement in total score of sexual function was used as dependent variable. The % improvements in frequency, urgency, nocturia, incontinence episodes, number of pads needed and voided volume were considered as independent variables. Data were analyzed using IBM Corp. Released 2016. IBM SPSS Statistics for Windows, Version 24.0. Armonk, NY: IBM Corp. Two-tailed *p* < 0.050 was considered significant.

## Results

All tested variables showed significant departures from the normal distribution. The only exceptions were the pre- and post-observation/treatment total scores of sexual function and the voided volumes. Consequently, non-parametric tests (Mann Whitney U test and Wilcoxon signed-rank test, respectively) were performed for all comparisons (for uniformity purposes) between and within groups, respectively; all data are presented as medians (interquartile ranges; IQRs).

Among the 48 women who reported sexual activity in the last 4 weeks from Group A, 11 women reported during the monthly visits that they had decided to follow pharmacotherapy/behavioral therapy for OAB and were therefore excluded from the study. Furthermore, 2 women from Group A and 2 women from Group B refused to complete the FSFI questionnaire at the end of 3 month period and were also excluded. There were no adverse reactions from mirabegron administration during the study.

Demographic characteristics and baseline OAB/sexual scores did not differ significantly between groups (Table 1). Sixty-five percent of patients in Group B who were incontinent at baseline became continent by the study-endpoint. Furthermore, all urinary outcomes, all FSFI domain scores and the total score of sexual function improved significantly in Group B at 3 months, in contrast to Group A (Tables 2, 3 and 4). Spearman’s rank-order correlation test yielded statistically significant (negative) correlations between % improvement in LUTS/incontinence and % improvement in sexual function were detected exclusively in Group B (frequency-lubrication, nocturia-arousal, incontinence-sexual satisfaction; Table 5).

**Table 1:** Demographic characteristics and baseline OAB/sexual scores of participants

Variable	Group A	Group B	*P* value
Age (yr)	43.5 (10.0)	43 (10)	0.838
Body weight (kg)	59.0 (14.0)	55.0 (14.0)	0.406
Symptom duration (yr)	4.2 (1.5)	4.2 (3.3)	0.821
Parity	2.0 (2.0)	2.0 (1.0)	0.738
Frequency	11.0 (1.0)	11.0 (2.0)	0.213
Urgency episodes	6.5 (1.0)	7.0 (2.0)	0.303
Nocturia episodes	2.0 (1.0)	2.0 (1.0)	0.227
Incontinence episodes	2.0 (1.0)	2.0 (1.0)	0.287
Incontinence pads	5.0 (2.0)	4.0 (2.0)	0.838
Voided volume (ml)	121.5 (31.0)	117.0 (33.0)	0.725
Desire	3.0 (0.6)	3.0 (1.2)	0.411
Arousal	3.6 (0.6)	3.0 (0.8)	0.281
Lubrication	3.6 (0.9)	3.9 (1.1)	0.214
Orgasm	3.6 (0.4)	3.6 (0.8)	0.253
Satisfaction	3.2 (0.4)	3.2 (0.8)	0.099
Pain	3.2 (0.4)	3.2 (0.8)	0.068
Total Score of Sexual Function	20.0 (2.5)	20.3 (3.8)	0.355

**Table 2:** Urinary evaluation of the participants

Group A	Pre-observation	Post-observation	*P* value
Frequency	11.0 (1.0)	11.0 (1.0)	0.200
Urgency episodes	6.5 (1.0)	7.0 (1.0)	0.093
Nocturia episodes	2.0 (1.0)	1.0 (1.0)	0.231
Incontinence episodes	2.0 (1.0)	2.0 (2.0)	0.332
Incontinence pads	5.0 (2.0)	5.0 (2.0)	0.231
Voided volume (ml)	121.5 (31.0)	114.5 (27.0)	0.001
Group B	Pre-treatment	Post-treatment	*P* value
Frequency	11.0 (2.0)	9.0 (2.0)	< 0.001
Urgency episodes	7.0 (2.0)	4.0 (2.0)	< 0.001
Nocturia	2.0 (1.0)	1.0 (1.0)	< 0.001
Incontinence episodes	2.0 (1.0)	1.0 (1.0)	< 0.001
Incontinence pads	4.0 (2.0)	2.0 (2.0)	< 0.001
Voided volume (ml)	117.0 (33.0)	148.0 (26.0)	< 0.001

**Table 3:** Comparison of pre-observation and post-observation FSFI in Group A

	Pre-observation	Post-observation	*P* value
Desire	3.0 (0.6)	3.0 (0.6)	0.524
Arousal	3.6 (0.6)	3.6 (0.6)	0.628
Lubrication	3.6 (0.9)	3.9 (0.6)	0.713
Orgasm	3.6 (0.4)	3.6 (0.4)	0.505
Satisfaction	3.2 (0.4)	2.8 (0.4)	0.109
Pain	3.2 (0.4)	3.2 (0.8)	0.424
Total Score of Sexual Function	20.0 (2.5)	19.8 (2.0)	0.609

**Table 4:** Comparison of pre-treatment and post-treatment FSFI in Group Β

	Pre-observation	Post-observation	*P* value
Desire	3.0 (1.2)	4.8 (1.2)	< 0.001
Arousal	3.0 (0.8)	4.8 (0.9)	< 0.001
Lubrication	3.9 (1.1)	4.8 (1.2)	< 0.001
Orgasm	3.6 (0.8)	4.8 (1.0)	< 0.001
Satisfaction	3.2 (0.8)	4.0 (0.8)	< 0.001
Pain	3.2 (0.8)	4.4 (1.2)	< 0.001
Total Score of Sexual Function	20.3 (3.8)	26.6 (4.2)	< 0.001

**Table 5:** Correlation between improvements (%) in lower urinary tract symptoms & female sexual function

	Desire		Arousal		Lubrication		Orgasm		Satisfaction		Pain		Total FSFI	
	r_s_	*P*	r_s_	*P*	r_s_	*P*	r_s_	*P*	r_s_	*P*	r_s_	*P*	r_s_	*P*
Group A														
Frequency	−0.088	0.553	0.220	0.298	− 0.201	0.171	− 0.237	0.104	0.015	0.918	− 0.105	0.479	− 0.254	0.081
Urgency	0.168	0.253	0.041	0.781	−0.195	0.199	−0.240	0.135	0.035	0.815	0.013	0.928	−0.224	0.127
Nocturia	0.209	0.154	0.157	0.286	−0.188	0.201	0.062	0.674	0.240	0.101	−0.054	0.714	0.181	0.219
Incontinence	−0.007	0.960	0.158	0.283	0.134	0.364	−0.270	0.064	0.101	0.496	−0.076	0.610	−0.026	0.858
Pads	−0.114	0.442	−0.076	0.607	−0.076	0.609	**−**0.180	0.114	0.119	0.422	−0.128	0.384	−0.222	0.150
Voided Volume	0.041	0.782	−0.067	0.651	−0.114	0.441	−0.010	0.948	0.228	0.119	0.126	0.393	0.077	0.603
Group B														
Frequency	0.103	0.545	−0.284	0.089	−0.429	0.008	0.139	0.413	−0.037	0.826	−0.230	0.170	−0.267	0.110
Urgency	0.005	0.977	−0.002	0.991	0.163	0.335	−0.119	0.484	0.253	0.132	0.011	0.946	0.008	0.638
Nocturia	−0.041	0.808	−0.352	0.032	−0.145	0.391	−0.062	0.716	0.161	0.340	0.080	0.640	−0.056	0.743
Incontinence	0.178	0.292	−0.336	0.042	−0.009	0.957	−0.209	0.214	0.250	0.130	0.206	0.221	0.068	0.687
Pads	0.123	0.468	−0.253	0.131	0.068	0.689	0.125	0.461	0.298	0.073	0.202	0.230	0.156	0.357
Voided Volume	0.186	0.271	−0.170	0.314	−0.074	0.663	0.269	0.108	0.205	0.223	0.161	0.340	0.177	0.294

Total score of sexual function improvement (%) differed significantly between groups (Group A vs. Group B: 1.0 (3.7) vs. 32.6 (7.9); *p* < 0.001). Multivariate linear regression analysis run to predict % improvement in total score of sexual function from group, as well as % improvements in frequency, urgency, nocturia, incontinence episodes, number of pads needed and voided volume revealed that Group and % improvements in frequency statistically significantly predicted % improvement in total score of sexual function, F(7, 77) = 141.970, *p* < .001, R^2^ = 0.928. All seven variables added statistically significantly to the prediction, *p* < 0.001. The multivariate linear regression model is presented in Table 6.

**Table 6:** Multivariate linear regression analysis model

Variable	B	SE B	β	*P* value	95.0% CI	
Constant	1.162	0.752		0.126	−0.336	2.660
Group	24.193	3.688	0.741	< 0.001	16.849	31.538
Frequency	−0.155	0.079	−0.107	0.050	−0.312	− 0.001
Urgency	0.003	0.049	0.005	0.951	−0.095	0.101
Nocturia	−0.001	0.012	−0.003	0.931	−0.025	0.023
Incontinence	0.004	0.018	0.009	0.843	−0.032	0.039
Number of pads	−0.015	0.031	−0.036	0.629	−0.077	0.047
Voided volume	0.113	0.065	0.123	0.086	−0.016	0.243

## Discussion

This study is one of the few, to our knowledge, to demonstrate the effect of mirabegron, the first β-3 adrenoceptor agonist, on sexual function of women suffering from OAB. Our data has indicated that after 3 months evaluation, females with OAB receiving mirabegron 50 mg revealed statistically significant changes in FSFI total score and all subscales.

OAB symptoms, urinary incontinence and sexual health in general are topics many find difficult to discuss. Not only may stigma make sufferers reluctant to approach a health professional, but it may also make health professionals embarrassed to confront patients. Self-reporting rather than interview-administered questionnaires greatly reduce this barrier. As FSFI allows a quantitative evaluation and can be used to study sexuality changes after therapeutic intervention, it has a distinct advantage over other assessment tools like diaries or calendars [24]. This tool is a reliable self-reporting measure of FSD which only requires 15 min to complete. Although it excludes issues involving personal stress, it measures outcomes of therapeutic response by design.

Female sexual dysfunctions can interfere with intimacy, affect a marital relationship and ultimately erode well-being and overall health. Although sexual dysfunction is more likely in women than men, clinical FSD trials are rare compared to the available burgeoning data on men. Furthermore, the amount of data related to the effect of treatment agents used for OAB on the sexual function of women is insufficient in the literature while study comparisons should be made with caution because of differences in study designs and population.

The use of anticholinergics as a first-line treatment for OAB is well documented. However, many large trials exclude sexual function change assessment after their administration. Tolterodine and oxybutynin are two anticholinergic medications with high quality evidence supporting improvement in sexual function with use. According to Hajebrahimi et al. [7], tolterodine IR significantly improved all domains of sexual function of women with OAB. The FSD was evaluated with the Arizona Sexual Experience Scale (ASEX), a five-topic questionnaire. Rogers et al. [25] evaluated the effect of short term treatment with tolterodine ER on FSD using the Pelvic Organ Prolapse/Urinary Incontinence Sexual Function Questionnaire (PISQ) and Sexual Quality of Life – Female (SQOL-F). OAB symptoms improved with tolterodine ER, as did the scores of sexual health and anxiety measures in sexually active women with OAB. Rogers et al. [26] revealed that in a population of racially diverse, sexually active women, long term tolterodine ER treatment for OAB resulted in a relief of symptoms as ascertained in bladder diaries. Their study also validated improved sexual health. Using three items from the King’s Health Questionnaire and one item from the Beck Depression Inventory, Sand et al. [27] showed that transdermal oxybutynin treatment for OAB improved sexual function. Young et al. [28], in a prospective study using the FSFI questionnaire, demonstrated that OAB symptom management using solifenacin had a positive outcome on female sexual function, especially on the domains of arousal, desire and satisfaction. On the other hand, according to Jha [29] treatment of OAB symptoms with anticholinergics, in female patients evaluated with the PISQ questionnaire, does not guarantee improvement in sexual health. The small sample size and the uncontrolled design of the studies may not permit the demonstration of a causal relationship between the regimen used for OAB treatment and FSD.

Other factors can have a greater effect on sexual life than urinary incontinence associated with intercourse. However, the enjoyment of intercourse can be adversely effected by urgency and frequency issues, along with the fear of leakage during stimulation and intercourse [30]. After 3 months of mirabegron treatment, results indicated statistically significant improvements in sexual function. These improvements may have played a critical role in overall women’s sexual health wellness, as revealed by total FSFI scores and individual sexual domain scores, in Group B. Thus, the improvement of FSFI scores, in the current study, may at least partly be attributable to the established improvements in urgency and frequency.

Numerous studies have demonstrated that sexual function is negatively affected in women with bladder dysfunction [6, 30]. It is obvious that the improvement in lower urinary tract symptoms results in improvement of women’s sexual life [7, 25–28]. These previous studies are consistent with our findings, demonstrating a positive influence of OAB symptom treatment with mirabegron on sexual health and quality of life. The fact that the number of incontinence pads used by patients was significantly reduced after treatment, might help women feel more desirable and willing to experience sexual relationships and may represent another mechanism to explain the improvement of FSFI demonstrated in the current study after mirabegron administration.

Extensive data exists on the *β*_3_-adrenergic receptor subtype of the sympathetic nervous system [31, 32]. In a recent study, *β*_3_-receptor stimulation caused rat aorta vasorelaxation via the activation of NO synthase and the associated increase of tissue levels of cGMP [33]. Gur et al. [34] demonstrated that mirabegron markedly relaxed isolated human corpus cavernosum (HCC) and rat corpora cavernosa by activating *β*_3_-adrenoceptors independently of the NO-cGMP pathway. Moreover, Cirino et al. [35] showed that the activation of the *β*_3_-receptors present in human corpus cavernosum elicits a cGMP dependent but NO-independent vasorelaxation which involves the inhibition of the RhoΑ/Rho-kinase pathway and facilitates erectile function.

An up-regulation of Rho kinase-β protein in alloxan-induced diabetic rabbit corporal tissue [36] and in the human corpus cavernosum [37] has recently been shown to control corporal smooth muscle contraction induced by endothelin-1. This suggests that the RhoA/Rho kinase pathway plays a mediatory role in increased sensitivity and force generation of corporal smooth muscle. Additionally, β3 receptor-mediated corporal smooth muscle relaxation involves the inhibition of RhoA/Rho-kinase [35]. Smooth muscle relaxation associated with phosphorylation and the resulting inhibition of RhoA is caused by the NO–cGMP signaling pathway mediated by the activation of the cGMP-dependent protein kinase [35, 38, 39]. As the cGMP-dependent protein kinase is found in human corpora cavernosa, this second mechanism is likely to occur in humans too. It seems, therefore, that the state of human corpus cavernosum tumescence may be regulated by the physiological function of these two pathways.

The clitoris is a complex structure that simulates the structure of penis, which is composed of two erectile bodies known as the corpora cavernosa. During sexual arousal, both the clitoris and the labia minora become engorged with blood. In addition, both vaginal and clitoral length and diameter increase. To date, the vascular contributions in FSD remain to be elucidated. The main blood supply to female genital tissue is via the internal pudendal artery (IPA) and clitoral artery (CA). A better understanding of female sexual arousal would be made possible if more was known about both these arteries. Allahdadi et al. [40] demonstrated that internal pudendal artery and clitoral artery are sensitive to the potent vasoconstrictor peptide, endothelin-1. Rho-kinase, however is a key component in endothelin-1 signaling. Furthermore, the inhibition of Rho-kinase in the internal pudendal artery and clitoral artery reduces ET-1-mediated constriction [40].

**Fig. 1:**
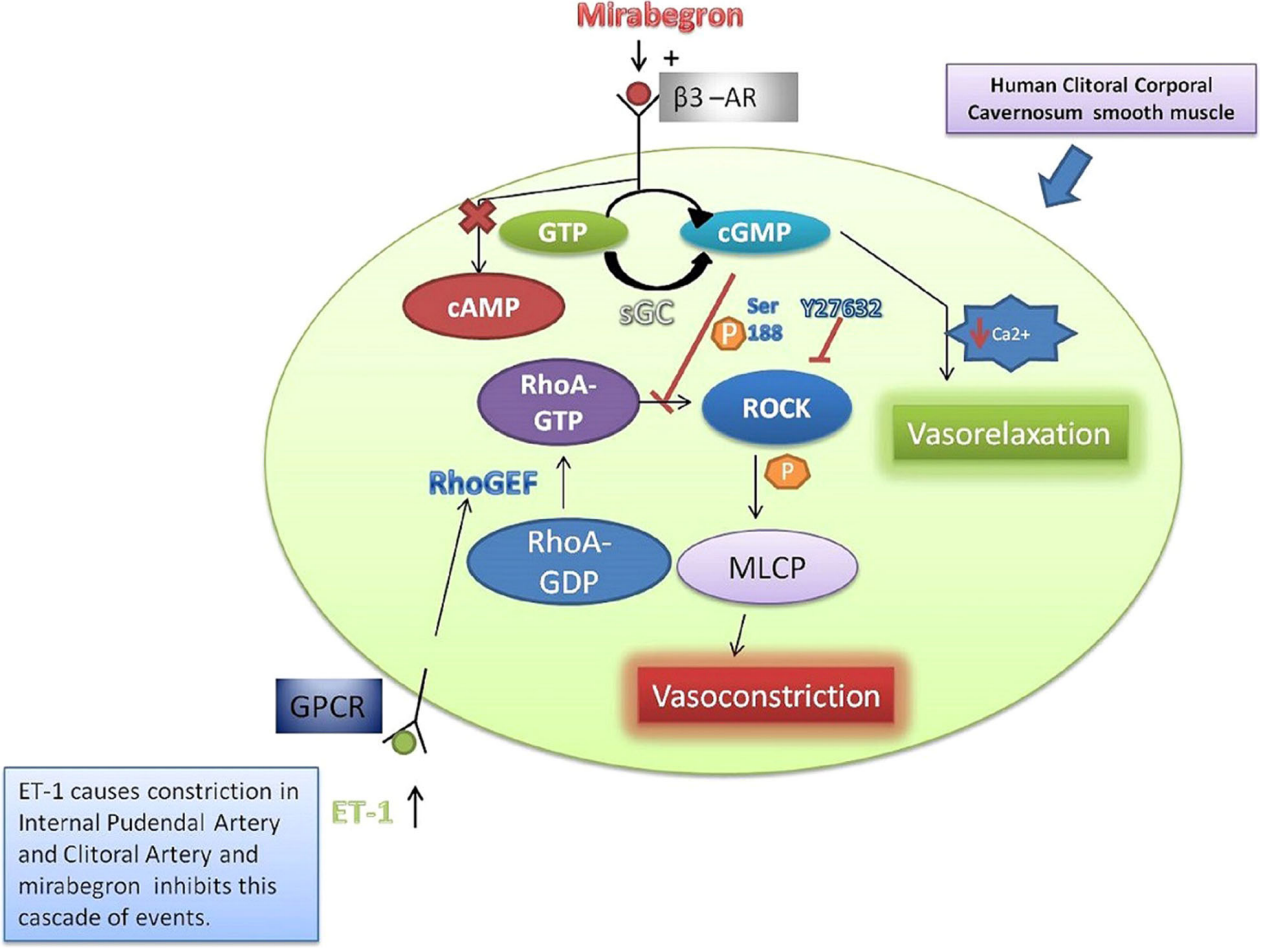
Possible mechanism of mirabegron’s effect, used for overactive bladder treatment, on female sexual function. β3- adrenergic receptor activation by mirabegron agonist is coupled to the generation of the second messenger cGMP, which causes human corporal cavernosum smooth muscle relaxation by lowering intracellular levels of free calcium. β3 receptor-mediated corporal smooth muscle relaxation involves inhibition of RhoA/Rho-kinase [34]. The Rho pathway is initiated by ET-1 agonist binding in the GPCR receptor, which activates RhoGEF, facilitating RhoA–GDP conversion to RhoA–GTP. RhoA–GTP binds to ROCK, facilitating autophosphorylation of ROCK that enhances its ability to phosphorylate and deactivate MLCP, promoting vasoconstriction. Relaxation is largely mediated by cGMP, which causes phosphorylation of RhoA, preventing interaction with ROCK and thereby inhibiting vasoconstriction. Endothelin-1-induced contraction of corporal smooth is mediated by an up-regulation of Rho kinase-β protein in alloxan-induced diabetic human corpus cavernosum [36]. Internal pudendal artery and clitoral artery are sensitive to the potent vasoconstrictor peptide, endothelin-1 (ET-1). The inhibition of Rho-kinase in the internal pudendal artery and clitoral artery reduces ET-1-mediated constriction [39].m. Abr. cGMP: cyclic GMP, GPCR: G-protein-coupled receptor, MLCP: myosin light chain phosphatase, RhoGEF: Rho guanine exchange factor, ROCK: Rho-associated protein kinase, sGC: soluble guanylyl cyclase, Y-27632: (*R*)-(+)-*trans*-N-(4-pyridyl)-4-(1-aminoethyl)-cyclohexanecarboxamide, ET-1: Endothelin-1

We may hypothesize that mirabegron, as a *β*_3_-adrenoreceptor agonist, induces relaxation of the corpus cavernosum that may enhance blood flow in the female region-clitoris which might be accompanied by increased stimulation. This proposed mechanism of mirabegron explains our results, in which mirabegron treatment caused statistically significant improvements in arousal, desire, orgasm and satisfaction female sexual function domains, as well as in total FSFI score (Fig. 1).

However, the present study has some limitations. Our study had a control group with women not taking mirabegron treatment but there is no placebo group. Thus, there is no evaluation about the effect of placebo on sexual function. It was not possible to use a randomization method since the decision of female patients to receive mirabegron or not, was the reason for being in certain group. Someone could claim that it is a random process of selection although that can by chance lead to disparities. In spite of these limitations, significant results have been obtained, which are of value for clinicians working in this field.

## Conclusions

Females with OAB should be assessed for their sexual function to provide better quality of life. According to the aforementioned data, OAB treatment with mirabegron improves female sexual function. The above documented improvement in female sexual function might be due to the improvement in the consequences of OAB pathophysiology. However an alternative mechanism may be raised attributing these beneficial effects in the women who received mirabegron treatment to mirabegron action per se. Our study is adherent to CONSORT guidelines.

## Abbreviations

ASEX: Arizona Sexual Experience Scale
BDI: Beck depression inventory
CA: Clitoral artery
cGMP: Cyclic Guanosine Monophosphate
ET-1: Endothelin-1
FSD: Female sexual dysfunction
FSFI: Female sexual function index
FSFI-Gr: Female sexual function index –greek
HCC: Human corpus cavernosum
ICS: International Continence Society
IPA: Internal pudendal artery
KHQ: King’s Health Questionnaire
NO: Nitric oxide
OAB: Overactive bladder
PISQ: Pelvic Organ Prolapse/Urinary Incontinence Sexual Function Questionnaire
SPSS: Statistical Package for the Social Sciences
SQOL-F: Sexual quality of life – female
Tolterodine ER: Tolterodine extended release
Tolterodine IR: Tolterodine immediate release
